# Persistence of a Cervical Neck Mass, Not Just the Lymphoma

**DOI:** 10.7759/cureus.746

**Published:** 2016-08-23

**Authors:** Sassine Ghanem, Mazen Zaarour, Uroosa Ibrahim, Samer Saouma, Ying Liu, Fanyi Kong, Jean Paul Atallah

**Affiliations:** 1 Internal Medicine, Staten Island University Hospital; 2 Hematology and Oncology, Tulane University; 3 Hematology/Oncology, Staten Island University Hospital; 4 Pathology and Laboratory Medicine, Staten Island University Hospital

**Keywords:** diffuse large b-cell lymphoma (dlbcl), actinomyces, cervicofacial actinomycosis, neck mass, lymphoma

## Abstract

Actinomycosis is a rare, chronic granulomatous infection caused by gram-positive, anaerobic to microaerophilic branching filamentous bacteria. In the cervicofacial region, it usually presents as an enlarging neck mass. It remains a diagnostic challenge due to the fact that cultures show no growth in more than 50% of cases. We report a case of a 67-year-old patient known to have a neck mass secondary to lymphoma in which the neck mass persisted despite therapy. Upon evaluation, the diagnosis of culture-negative actinomycosis was based on histopathology findings, and the patient received antibiotic therapy. We will discuss the diagnosis and pathology of actinomycosis, attempting to explore the relationship between actinomycosis and lymphoid malignancy.

## Introduction

The oral cavity is not a sterile site and is constantly colonized by commensal flora that may become pathogenic in the right setting. *Actinomyces *are gram-positive, anaerobic or microaerophilic, non-spore-forming, branched rods that make up part of this flora. Rarely, the mucocutaneous barrier is breached, and this leads to actinomycosis. Infection with actinomycosis is rarely driven by the *Actinomyces *species alone but usually occurs with synergistic bacteria. The rapidity of the clinical evolution depends on the type of synergistic bacteria, and therefore, it can present as acute or chronic. The acute form is characterized by a painful abscess or a phlegmonous cellulitis. The chronic form manifests as a painless neck mass composed of multiple abscesses and draining sinus tracts of pus [1]. Taking into consideration the difficulty in the culture of *Actinomyces*, it is the chronic form of a painless neck mass for which actinomycosis deserves being referred to as the great mimicker.

## Case presentation

The patient is a 67-year-old gentleman who presented to the oncology clinic in September of 2015 with a non-tender, left neck swelling. The patient had lost approximately 8 kgs over the previous six months. He denied any history of fever, chills, dysphagia, recent dental procedure, or trauma to the area. A CT scan of the neck showed a heterogeneous mixed cystic solid mass 3.2 x 4.5 x 4.0 cm adjacent to the left internal jugular lymph nodes. A fine needle aspiration and core biopsy of the mass showed a monomorphic population of large lymphoid cells. Biopsy and flow cytometry findings were consistent with diffuse, large B-cell lymphoma. Post-procedure, the patient developed a worsening of the neck swelling. Investigation with a CT scan of the cervical region showed interval development of a soft tissue hematoma with no evidence of active bleeding. The patient agreed to participate and was explained the nature and objectives of this study, and informed consent was formally obtained. No reference to the patient's identity was made at any stage during data analysis or in the report.

**Figure 1:**
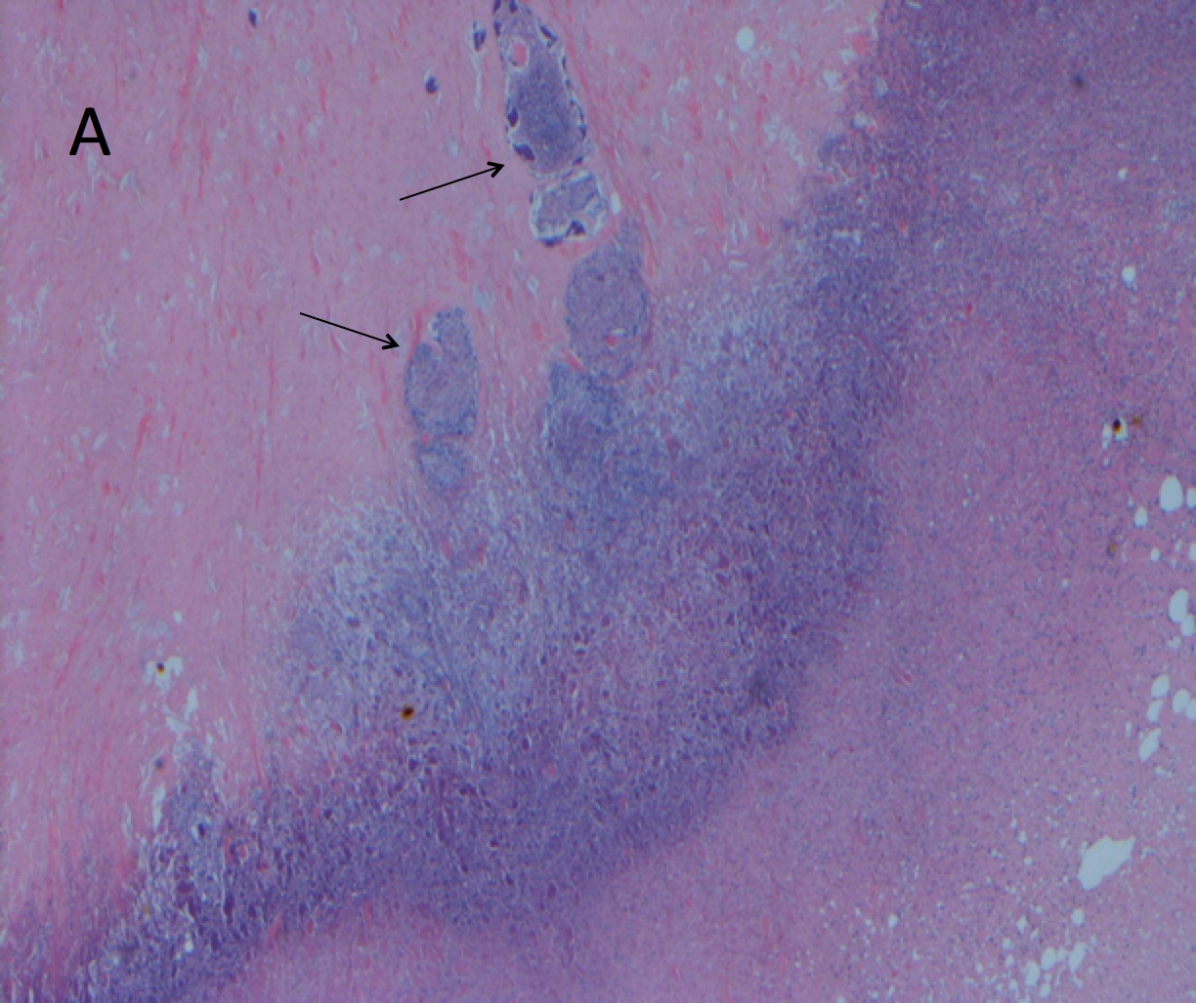
Pathology Showing Extensive Necrosis with Actinomyces Clones with Adjacent Suppurative Granuloma H&E stain, X100 magnification.

**Figure 2:**
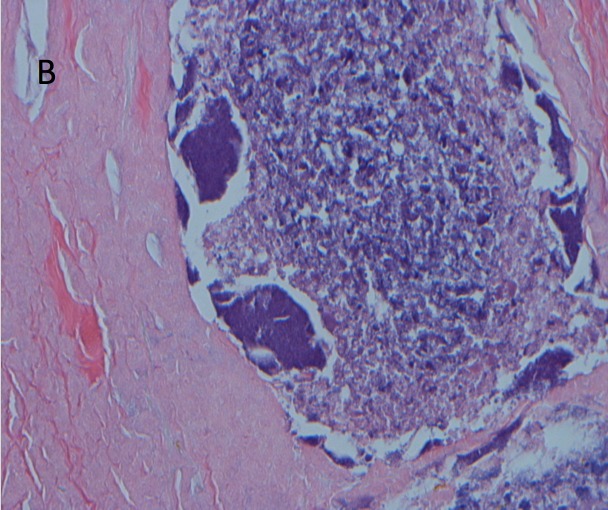
Pathology Slide Showing Lumps of Basophilic Bacteria in a Vaguely Rosette-Like Configuration and Adjacent Granuloma with Central Abscess Formation H&E stain, X400 magnification

The patient was started on chemotherapy with dose-adjusted R-EPOCH (rituximab, etoposide, prednisone, vincristine, cyclophosphamide, adriamycin). He was treated for three cycles without any adverse events. Nevertheless, he continued to have low-grade fever and no significant decrease in the size of the neck mass. Four months after the initial diagnosis, a repeat CT of the neck was done that showed persistence of the neck hematoma, now 5.8 cm in greatest dimension (previously 7.6 cm), with a new peripheral rim enhancement suspicious for an infectious process. The fluid portion of the mixed mass was therefore aspirated under ultrasound guidance, and the patient was started on empirical antibiotic therapy with Clindamycin and Ampicillin/Sulbactam. The cultures of the aspirated fluids, including aerobic, anaerobic, mycobacterial, and fungal-specific media, were all negative for growth. The patient continued to spike a fever despite broad-spectrum empiric therapy. This prompted an otolaryngology evaluation for excision of the mass. The patient underwent a modified radical neck dissection with sparing of the internal jugular vein, sternocleidomastoid muscle, and spinal accessory nerve. The histopathology was consistent with extensive necrosis containing *Actinomyces* clones with adjacent suppurative granuloma (Figures 1-2).

Of note, Gram, Ziehl–Neelsen, Grocott's methenamine silver, and Periodic acid–Schiff stains of the fluid taken from the surgical procedure were all negative. The patient was treated with ampicillin-sulbactam 3 mg intravenously every six hours for a total of eight weeks. The abscess cleared after completion of antibiotic therapy. He completed his fourth cycle of chemotherapy and remains in remission to date.

## Discussion

Actinomycosis is an uncommon, suppurative and granulomatous infection caused by *Actinomyces* species. The causative microorganisms are gram-positive, anaerobic or microaerophilic, non-spore-forming branched rods. The sole reservoir of pathogenic *Actinomyces* is humans, and they belong to the commensal flora of the oropharynx, gastrointestinal, and urogenital tract [1].

Infection occurs in all age groups but rarely affects children or the elderly (older than 60 years of age). The male to female ratio is 3 to 1. The cervicofacial region is involved in 60% of cases, as compared to 15% and 20% for thoracic and abdominal regions, respectively [1]. The patient in our case had cervicofacial actinomycosis, therefore our discussion will focus on this form of the disease.

Despite having low pathogenicity, the *Actinomyces* species can cause disease when there is disruption of the mucocutaneous barrier. The inciting factor for cervicofacial actinomycosis is mainly odontogenic in origin. Risk factors include poor dental hygiene, dental manipulations, immunosuppression, oromaxillofacial trauma, and chronic head and neck infections [2]. *Actinomyces Israelii *and *Actinomyces Gerencseriae *comprise almost 70% of cases. Moreover, most of the actinomycotic lesions contain co-pathogens that favor the spread of the infection [1]. Of the aforementioned risk factors, the patient in our case had only poor dental hygiene.

The composition of the synergistic flora usually determines the rapidity of onset of the actinomycosis. The infection may be acute or chronic. The acute infection is characterized by a painful abscess or a phlegmonous cellulitis. The chronic form has a more insidious course and manifests as a painless induration with multiple abscesses and draining sinus tracts of pus [1]. Although hematogenous dissemination can occur, lymphatic spread is uncommon due to the size of the bacterium, and therefore, lymphadenopathy is typically absent [3].

Actinomycosis is often a diagnostic challenge since it can mimic numerous infectious and non-infectious conditions. The definitive diagnosis is confirmed only following the isolation of *Actinomyces* from a sterile site [4]. This can be difficult oftentimes because of inappropriate culture conditions (including short incubation), previous or concomitant antibiotic therapy, and or inhibition of *Actinomyces* growth by other bacteria [5]. A gram stain from pus or tissue specimens shows branching gram-positive filamentous rods, while cultures require an anaerobic media and are sterile in more than 50% of cases [4]. Another important, yet non-specific, histological clue for the diagnosis is the presence of sulfur granules in tissue specimens or drainage. These granules are yellow aggregates of bacterial colonies [4]. In our case, the diagnosis was difficult to establish since the cultures were repeatedly negative. The fine needle aspiration (FNA) biopsy of the mass performed several months prior to diagnosis failed to show any *Actinomyces*. The yield on FNA is low, however, for the disease; therefore, it is unclear if the infection was missed at the initial presentation or whether it developed later.

The mainstay of treatment for this infection is Penicillin G, with or without surgical intervention. There is no clear consensus regarding the duration of therapy, which can range between two and twelve months [6]. In fact, a prolonged course is often needed to overcome the fibrotic tissue that results from the granulomatous inflammation. Shorter courses of treatment with complete response have been reported in the cervicofacial region in two cases by Sudhakar et al [7]. If a known pathogen is found concomitantly, the tendency is usually to treat this pathogen as well. Indications for surgical treatment include sinus tracts or fistula formation, extensive fibrosis, or suspicion for a different granulomatous process or malignancy [4].

The possible association between actinomycosis and lymphoma is a known, yet debatable subject. The exact underlying mechanism for such a postulated relation remains unclear. A possible hypothesis is that the malignancy-induced damage to the underlying tissues (with reduced oxygenation) will serve as a port of entry for this anaerobic bacterium. This is corroborated by the failure of resolution of the infection with antimicrobials alone, as demonstrated in several cases in the literature (Table 1). Another hypothesis to be considered is the alteration of local defenses and the immunocompromised state associated with malignancy, predisposing patients to opportunistic infections, which in this case would be actinomycosis.

**Table 1:** The Variable Response to Antibiotic and Chemotherapy in Reported Cases of Actinomycosis and Lymphoma Co-Occurrence

Cases	Site of Infection	Type of Lymphoma	Treatment Outcome
Winter et al., (1983) [8]	Thoracic	Lymphocytic lymphoma	- No initial response to prolonged antibiotic course (4 months with 2 different drugs). - Infection only improved after initiation of chemotherapy.
Guerci et al., (1996) [9]	Thoracic	Abdominal follicular non-hodgkins lymphoma (NHL)	- Initial response to antibiotics followed by recurrence. - Lymphoma diagnosed years after the initial infection, with good response following chemotherapy.
Guerci et al., (1996) [9]	Abdominal (Hepatic)	Abdominal and thyroid NHL	- Lymphoma diagnosed 6 weeks after initiation of antibiotics. - Favorable response to simultaneous antibiotic and chemotherapy.
Batt et al., (1996) [10]	Pulmonary	Thoracic NHL	- Infection isolated years after the diagnosis of lymphoma. - Favorable response to simultaneous antibiotic and chemotherapy.

The patient in our case was diagnosed with diffuse large B-cell lymphoma several months prior to the isolation of the *Actinomyces*. The diagnosis of the infection was only made after he presented with persistence of the neck mass and fever. In this setting, clinicians should be aware of the hazards of anchoring on an initial diagnosis in case of lack of response to therapy, and a repeat biopsy is indicated to rule out a superimposed infection. 

## Conclusions

Although actinomycosis and lymphoma are known to have some association, only a limited number of cases have been reported in the literature to support the coexistence of these two entities. A high index of suspicion is warranted for the diagnosis since improvement of the clinical course of the infection in some patients only occurs following the treatment of both conditions. A clinician should, therefore, always have infection on his differential diagnosis when faced with a non-resolving malignant mass despite adequate therapy. 
